# Optimal Anticoagulant Strategy for Periprocedural Management of Atrial Fibrillation Ablation: A Systematic Review and Network Meta-Analysis

**DOI:** 10.3390/jcm11071872

**Published:** 2022-03-28

**Authors:** Tabito Kino, Minako Kagimoto, Takayuki Yamada, Satoshi Ishii, Masanari Asai, Shunichi Asano, Hideto Yano, Toshiyuki Ishikawa, Tomoaki Ishigami

**Affiliations:** 1 Cardiovascular Research Center, Lewis Katz School of Medicine, Temple University, Philadelphia, PA 19140, USA; tabito.kino@temple.edu; 2 Department of Cardiology, Kanagawa Cardiovascular and Respiratory Center, Yokohama 236-0051, Japan; minako.kagimoto@gmail.com (M.K.); e103004g@yokohama-cu.ac.jp (S.I.); 3 Department of Medicine, University of Pittsburgh, Pittsburgh, PA 15261, USA; yamadat@upmc.edu; 4 Department of Cardiology, Saiseikai Yokohamashi Nanbu Hospital, Yokohama 234-0054, Japan; masanari@yokohama-cu.ac.jp; 5 Department of Cardiology, Yokohama Rosai Hospital, Yokohama 222-0036, Japan; shunasncirc@gmail.com; 6 Department of Cardiology, Gyotoku General Hospital, Ichikawa 272-0103, Japan; yanohi@juno.dti.ne.jp; 7 Department of Medical Science and Cardiorenal Medicine, Yokohama City University Graduate School of Medicine, Yokohama 236-0004, Japan; tishika@yokohama-cu.ac.jp

**Keywords:** periprocedural anticoagulant management, atrial fibrillation ablation, direct oral anticoagulant, vitamin-K antagonist, network meta-analysis

## Abstract

This network meta-analysis was performed to rank the safety and efficacy of periprocedural anticoagulant strategies in patients undergoing atrial fibrillation ablation. MEDLINE, EMBASE, CENTRAL, and Web of Science were searched to identify randomized controlled trials comparing anticoagulant regimens in patients undergoing atrial fibrillation ablation up to July 1, 2021. The primary efficacy and safety outcomes were thromboembolic and major bleeding events, respectively, and the net clinical benefit was investigated as the primary-outcome composite. Seventeen studies were included (*n* = 6950). The mean age ranged from 59 to 70 years; 74% of patients were men and 55% had paroxysmal atrial fibrillation. Compared with the uninterrupted vitamin-K antagonist strategy, the odds ratios for the composite of primary safety and efficacy outcomes were 0.61 (95%CI: 0.31–1.17) with uninterrupted direct oral anticoagulants, 0.63 (95%CI: 0.26–1.54) with interrupted direct oral anticoagulants, and 8.02 (95%CI: 2.35–27.45) with interrupted vitamin-K antagonists. Uninterrupted dabigatran significantly reduced the risk of the composite of primary safety and efficacy outcomes (odds ratio, 0.21; 95%CI, 0.08–0.55). Uninterrupted direct oral anticoagulants are preferred alternatives to uninterrupted vitamin-K antagonists. Interrupted direct oral anticoagulants may be feasible as alternatives. Our results support the use of uninterrupted direct oral anticoagulants as the optimal periprocedural anticoagulant strategy for patients undergoing atrial fibrillation ablation.

## 1. Introduction

Atrial fibrillation (AF) is a cardiac arrhythmia common worldwide [1]. Catheter ablation (CA) is the most effective treatment to prevent AF recurrence [2], and over the last decade, it has resulted in dramatic improvements in safety and efficacy [3,4,5,6,7]. However, periprocedural complications occur in approximately 4–14% of patients undergoing AF ablation, 2–3% of which are potentially life-threatening [8]. Periprocedural stroke or transient ischemic attack (TIA) and cardiac tamponade are the most notable complications [9,10]. As these adverse events are affected by periprocedural anticoagulant management, an optimal anticoagulant strategy is essential for the prevention of thromboembolic and bleeding complications.

Compared with vitamin-K antagonists (VKAs), direct oral anticoagulants (DOACs) have been shown to have a favorable risk-benefit profile, as they significantly reduce the incidence of stroke and also carry a similar bleeding risk in the long-term treatment of patients with AF [11]. With respect to CA, many studies have found that DOACs have similar efficacy and safety compared with VKAs [12,13,14,15,16,17,18,19]. These results led to the guideline recommendation of uninterrupted anticoagulants for the perioperative management of patients undergoing AF ablation [8,9]. Conversely, a German survey reported that interrupted and minimally interrupted DOAC was used more frequently than truly uninterrupted DOAC to avoid bleeding complications [20]. Moreover, some meta-analyses including randomized controlled trials (RCTs) have revealed that interrupted DOAC was not inferior to uninterrupted DOAC administration and was preferable to uninterrupted VKA administration [21,22]. Currently, guidelines lack indications based on these RCTs regarding which strategy is preferable for periprocedural anticoagulant management. This was the first study comparing each strategy and regimen with network meta-analysis (NMA) to simultaneously compare multiple treatments in a single analysis by combining direct and indirect evidence within a network of RCTs [23].

This study aimed to synthesize the available evidence from RCTs using NMA to: (1) assess the relative effects of different uninterrupted or interrupted anticoagulant strategies between DOACs and VKAs for reducing thromboembolic or bleeding events in patients undergoing AF ablation; and (2) to rank regimens, uninterrupted or interrupted, and DOAC or VKA administration for effectiveness in preventing thromboembolic or bleeding complications.

## 2. Materials and Methods

### 2.1. Protocol and Registration

Our report follows the preferred reporting items for systematic reviews and meta-analyses (PRISMA)-NMA extension (Table S1) [24]. The study protocol was registered at PROSPERO (CRD42021268787).

### 2.2. Eligibility Criteria

Only studies that met the eligibility criteria were included. The criteria were: (1) only RCTs; (2) uninterrupted or interrupted anticoagulant strategy in the periprocedural period; (3) patients undergoing AF ablation; and (4) publication of efficacy (stroke, TIA, or systemic embolism) and safety (major bleeding) outcomes. We excluded duplicate studies. There were no language or publication date restrictions.

### 2.3. Search Strategy

We performed a systematic search of the MEDLINE, EMBASE, Cochrane Central Register of Controlled Trials, and Web of Science databases up to July 1, 2021. The search used the Population, Intervention, Comparators, Outcomes, and Study design format and included the following terms: atrial fibrillation, ablation, periprocedural anticoagulation, and randomized controlled trial (Table S2). Three independent and blinded reviewers (SI, MA, and SA) separately assessed the search results to select studies based on the eligibility criteria. When a consensus was not reached by the three reviewers, a fourth author (TI) was consulted to reach a decision.

### 2.4. Outcomes

The primary efficacy outcome was thromboembolic events, including stroke, TIA, or systemic embolism. The primary safety outcome was major bleeding as defined by the Bleeding Academic Research Consortium (BARC) [25] or the International Society on Thrombosis and Hemostasis (ISTH) [26]. The secondary safety outcome was minor bleeding, and the secondary efficacy outcome was asymptomatic cerebral embolism (ACE). ACE was diagnosed using diffusion-weighted magnetic resonance imaging (MRI). Minor bleeding was defined as any bleeding that did not fulfil the BARC or ISTH criteria. The net clinical benefit was investigated as a composite of the primary safety and efficacy outcomes.

### 2.5. Data Extraction and Synthesis

We extracted the following data from the studies: study name, baseline characteristics, anticoagulant regimens, and outcomes. Two reviewers (MK and TY) independently extracted the data. When disagreements between reviewers occurred, a third author (TI) was consulted to reach a decision.

All study regimens were synthesized as follows: uninterrupted DOAC (UI-DOAC), interrupted DOAC (I-DOAC), uninterrupted VKA (UI-VKA), and interrupted VKA (I-VKA) administration. The number of thromboembolic events, major bleeding, composite of primary outcomes, minor bleeding, and ACE were synthesized, and odds ratios (ORs) were estimated. Additionally, all strategies were synthesized per anticoagulant (apixaban, dabigatran, edoxaban, rivaroxaban, and warfarin), and the ORs of the composite of the primary outcomes were estimated in subgroup analyses. The geometry of the network was illustrated using direct comparative treatments.

### 2.6. Risk of Bias Assessment

We evaluated the risk of bias using the revised Cochrane risk-of-bias tool for randomized trials (RoB2) [27]. Two reviewers (MK and TY) were involved in the quality assessment; if disagreements occurred, a third author (TI) was consulted to reach a consensus.

### 2.7. Statistical Analysis

NMA statistical analyses were performed with frequentist methods using *Netmeta* (version 1.5-0) in R 4.1.0 (R Foundation for Statistical Computing, Vienna, Austria). The ORs and 95% confidence intervals (CIs) were estimated based on a random effects model. Additionally, we calculated the P-score and the surface under the cumulative ranking (SUCRA) to evaluate and rank the anticoagulant strategies and regimens [28,29]. Both rankings are measured on a scale from 0 (worst) to 1 (best). Common network heterogeneity was evaluated using the *I*^2^ measure to locate the source of heterogeneity [30]. Heterogeneity was defined as low, moderate, or high when *I*^2^ was 25%, 50%, or 75%, respectively [31]. Inconsistency between direct and indirect evidence was examined globally and locally [32,33]. Begg’s rank correlation and Egger’s linear regression were performed to assess publication bias among the studies [34,35]. We conducted sensitivity analyses by excluding one study at a time for the four different strategies of the network. Subgroup analysis was performed to evaluate each anticoagulant regimen for the composite of the primary outcomes.

## 3. Results

### 3.1. Study Identification and Study Population Characteristics

**Figure 1:**
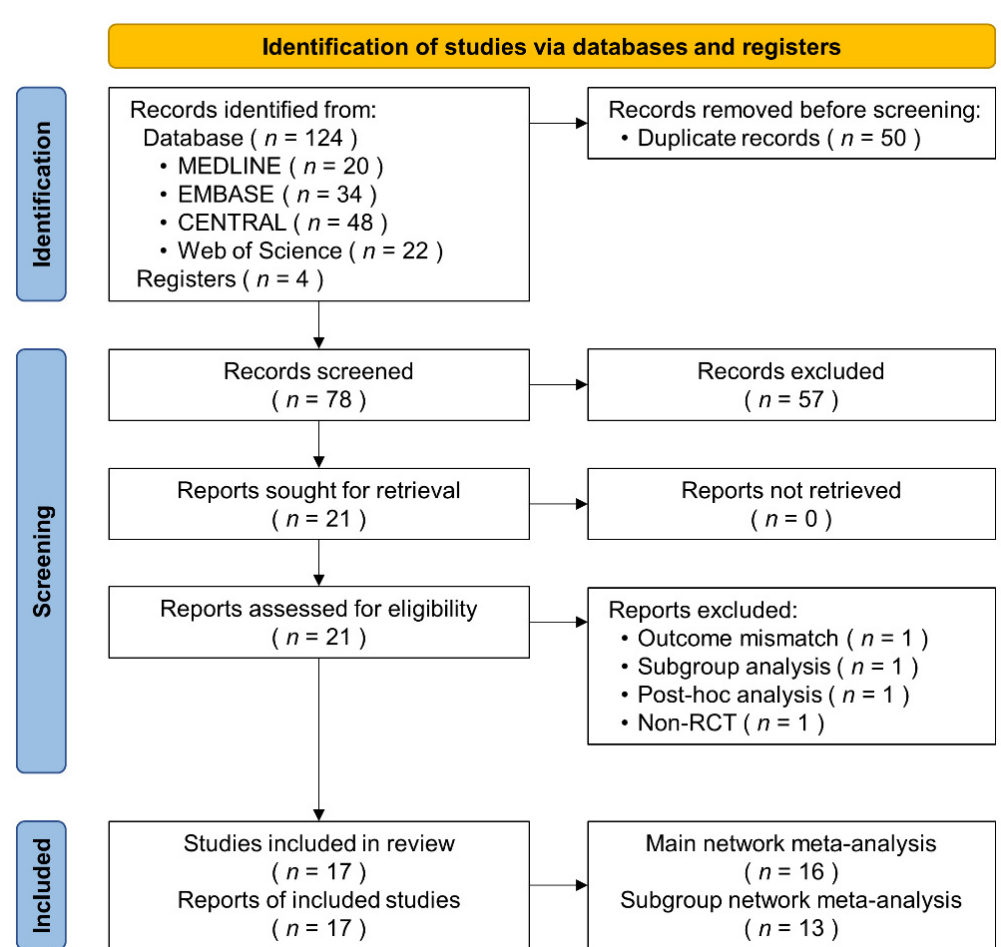
Flow diagram of the included studies. The PRISMA flow diagram depicts the phases of the systematic review and shows the number of records identified, screened, included, and excluded. RCT, randomized controlled trial.

We initially identified 124 studies via the electronic databases, and four additional studies were identified through references. Fifty duplicate studies were removed and 78 were screened. We excluded 57 studies after screening the titles and abstracts, and 21 were retrieved for detailed evaluation, from which four studies were subsequently excluded from the analysis because they did not meet the eligibility criteria (Figure 1). Finally, our meta-analysis included 17 RCTs with 6950 patients undergoing AF ablation [36,37,38,39,40,41,42,43,44,45,46,47,48,49,50,51,52]. They were allocated to I-VKA (*n* = 835) [36,37], UI-VKA (*n* = 2097) [36,38,39,40,41,42,43,44], I-DOAC (*n* = 1465) [37,38,45,46,47,48,49,50,51], and UI-DOAC (*n* = 2553) groups [39,40,41,42,43,44,45,46,47,48,49,50,51,52]. All approved DOACs (apixaban [40,43,45,46,47,48,49,50,51], dabigatran [37,38,41,47,48,51], edoxaban [44,48,49,51,52], and rivaroxaban [39,42,45,47,48,49,51,52]) were included.

The age of the participants ranged from 59 to 70 years (median = 62 years); 74% were men and 55% had paroxysmal AF. Twelve studies (71%) reported mean CHA_2_DS_2_-VASc scores ranging from 1.5 to 2.8 (median 2.0). In 15 studies (88%), the median follow-up duration was 30 days (range: 2–90 days). The detailed clinical characteristics of the included studies are summarized in Table 1 and Table S3.

All studies reported primary efficacy (thromboembolic events) and safety (major bleeding) outcomes, while 14 and 8 studies (82% and 47%, respectively) reported minor bleeding [36,37,38,39,40,41,42,43,44,46,48,49,50,51] and ACE [40,42,43,44,45,48,49,52], respectively.

### 3.2. Risk of Bias Assessment

We evaluated all studies in five dimensions (Table S4). Concerns were noted for 14 RCTs (82%). All protocols were composed of two interventions after randomization: anticoagulant initiation and AF ablation. Since some deviations occurred before CA, outcomes were analyzed as a modified intention-to-treat population who underwent AF ablation. Some small RCTs did not mention the concealment method. However, none of the studies were classified as having a high bias risk. Therefore, we included all studies in the NMA.

### 3.3. Structure of the Network

**Figure 2:**
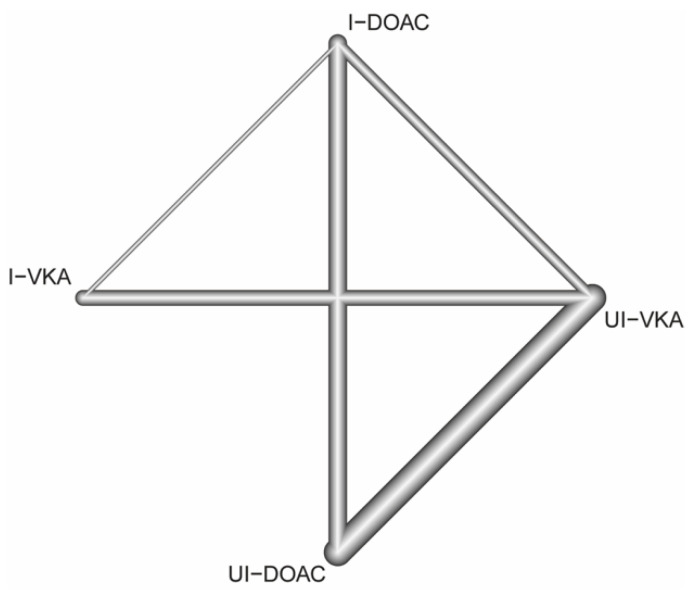
Network of treatment comparisons for the overall primary efficacy and safety outcomes. Directly comparable treatments are linked to lines. The nodes are placed and labelled according to the treatments. The thickness of the edges is proportional to the inverse standard error of the treatment effects, aggregated over all studies, including the two respective treatments. The network includes 16 two-armed studies. UI, uninterrupted; I, interrupted; DOAC, direct oral anticoagulant; VKA, vitamin-K antagonist.

Figure 2 shows the network of anticoagulant strategies used in the main analysis. We compared four strategies: UI-DOAC, I-DOAC, UI-VKA, and I-VKA, and set UI-VKA as a reference. All direct comparative studies were included, except UI-DOAC vs. I-VKA.

### 3.4. NMA Results for the Primary and Secondary Outcomes

**Figure 3:**
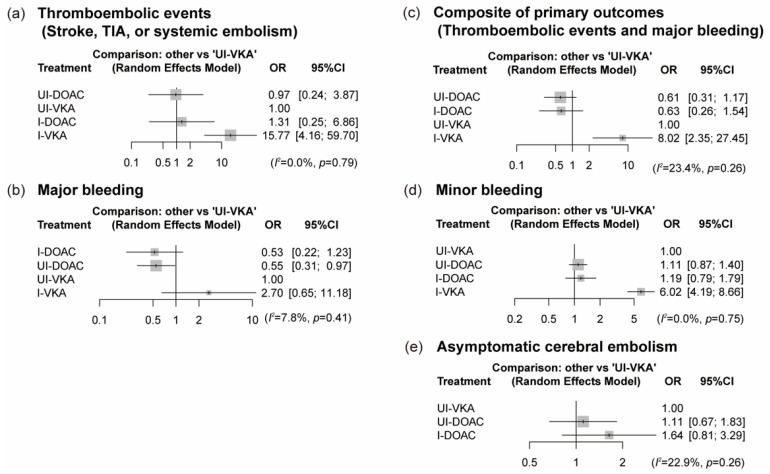
Forest plots for efficacy and safety of anticoagulant strategies compared with UI-VKA. (**a**) The efficacy of thromboembolic events (stroke, TIA, or systemic embolism); (**b**) the safety of major bleeding; (**c**) the efficacy and safety of the composite of the primary outcomes (stroke, TIA, or systemic embolism and major bleeding); (**d**) the safety of minor bleeding; and (**e**) the efficacy of asymptomatic cerebral embolism. TIA, transient ischemic attack; OR, odds ratio; CI, confidence interval; UI, uninterrupted; I, interrupted; DOAC, direct oral anticoagulant; VKA, vitamin-K antagonist.

The results of the NMA for thromboembolic events, major bleeding, and the composite of primary outcomes are presented as forest plots (Figure 3a–c). I-VKA was associated with an increased risk of thromboembolic events compared to UI-VKA (OR [95% CI]: 15.77 [4.16–59.70]), whereas there was no significant difference between UI-DOAC and I-DOAC (OR: 0.97 [0.24–3.87] and OR: 1.31 [0.25–6.86]). Compared to UI-VKA, UI-DOAC significantly decreased the risk of major bleeding (OR: 0.55 [0.31–0.97]). However, I-DOAC and I-VKA did not have a significant effect (OR: 0.53 [0.22–1.23] and OR: 2.70 [0.65–11.18]). In the composite of thromboembolic events and major bleeding, UI-DOAC and I-DOAC were not inferior to UI-VKA (OR: 0.61 [0.31–1.17] and OR: 0.63 [0.26–1.54]), while I-VKA significantly increased the risk of thromboembolic events and major bleeding (OR: 8.02 [2.35–27.45]). Heterogeneity was low for primary outcomes (thromboembolic events, *I*^2^ = 0.0%; major bleeding, *I*^2^ = 7.8%; and the composite of primary outcomes, *I*^2^ = 23.4%).

Regarding the secondary outcomes, the NMA results for minor bleeding and ACE are presented in Figure 3d,e. Both UI-DOAC and I-DOAC carried comparable risks of minor bleeding with UI-VKA (OR: 1.11 [0.87–1.40] and OR: 1.19 [0.79–1.79]), but I-VKA significantly increased the risk (OR: 6.02 [4.19–8.66]). UI-DOAC and I-DOAC were also similar to UI-VKA for the risk of ACE (OR: 1.11 [0.67–1.83] and OR: 1.64 [0.81–3.29]). Heterogeneity was low for secondary outcomes (minor bleeding, *I*^2^ = 0.0%; and ACE, *I*^2^ = 22.9%).

Table 2 displays the P-score and SUCRA values for the primary and secondary outcomes. There were no ranking mismatches between the P-score and SUCRA. The SUCRA value of DOACs was nearly twice that of UI-VKA in the composite of the primary outcomes (UI-DOAC, 0.82; I-DOAC, 0.77; and UI-VKA, 0.40). In contrast, the secondary outcome SUCRA values were higher for UI-VKA than for DOACs; particularly, the ACE value for I-DOAC was markedly low (UI-DOAC, 0.60; I-DOAC, 0.09; and UI-VKA, 0.82).

Overall, the UI-DOAC strategy was favorable and the I-DOAC strategy was feasible compared with the UI-VKA strategy for the primary and secondary outcomes. However, the I-VKA strategy significantly increased the risk of thromboembolic and bleeding events compared to the UI-VKA strategy.

### 3.5. Sensitivity Analyses

We performed sensitivity analyses for the composite of the primary outcomes (Table S5). RE-CIRCUIT and ELIMINATE-AF were the main sources of heterogeneity [41,44]. Moreover, UI-DOAC was associated with a significant reduction in major bleeding; however, this finding was not robust because it was no longer significant when 10 studies were excluded. Overall, this analysis did not suggest that the excluded studies would affect the relative effects and rankings of the anticoagulant strategies.

### 3.6. Assessment of Inconsistency and Publication Bias

The inconsistency test did not suggest the presence of inconsistency in the network (Figure S1). Begg’s and Egger’s tests did not reveal significant publication bias among the included studies (Figure S2).

### 3.7. Subgroup Analysis

**Figure 4:**
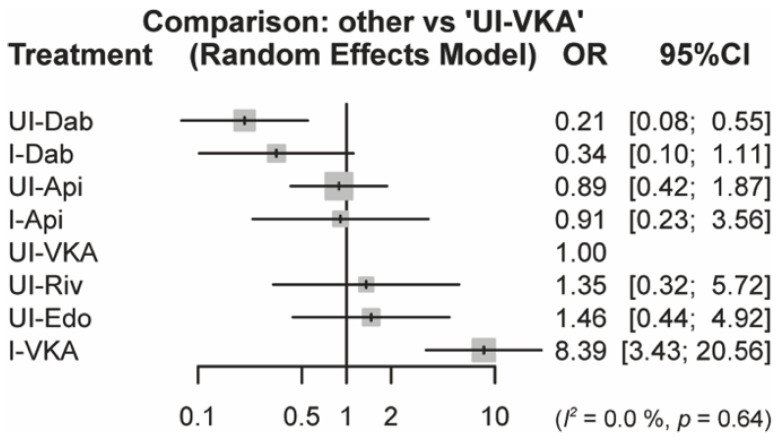
Forest plot for the composite of primary outcomes for each anticoagulant regimen. OR, odds ratio; CI, confidence interval; VKA, vitamin-K antagonist; UI, uninterrupted; I, interrupted; Dab, dabigatran; Api, apixaban; Riv, rivaroxaban; Edo, edoxaban.

We conducted a subgroup analysis based on each anticoagulant regimen. The composite of thromboembolic and major bleeding events was analyzed and displayed in a forest plot (Figure 4) and a league table (Table S6); we also calculated the P-score and SUCRA values (Table 3). Four studies were excluded because they had no randomized regimens for each DOAC [47,48,49,51], and one study included both UI-DOAC arms [52]. The structure of the subgroup network is shown in Figure S3. UI-dabigatran significantly decreased the risk of the composite of the primary outcomes compared with UI-VKA (OR: 0.21 [0.08–0.55]), whereas I-VKA significantly increased the risk (OR: 8.39 [3.43–20.56]). Other anticoagulants had a comparable risk to that of UI-VKA. The P-score and SUCRA values were notably higher for UI-dabigatran and I-dabigatran than for the other anticoagulant regimens (UI-dabigatran, 0.95; and I-dabigatran, 0.82 in SUCRA).

## 4. Discussion

In this study, we compared uninterrupted or interrupted DOAC administration with uninterrupted or interrupted VKA administration as periprocedural anticoagulant strategies for patients undergoing AF ablation. The main findings were as follows: (1) the risk of thromboembolic events among the strategies was exceedingly rare (UI-DOAC: 0.20%, I-DOAC: 0.20%, and UI-VKA: 0.24%) and not significantly different within strategies, except for the I-VKA strategy (4.79%); (2) ACE occurred with an incidence of 15–21%; (3) major bleeding tended to be halved by DOAC compared with UI-VKA administration; (4) minor bleeding did not differ between DOACs and VKAs, except for I-VKA; and (5) UI-dabigatran significantly reduced the composite of thromboembolic and major bleeding events.

After the COMPARE trial [36], the UI-VKA strategy has been widely adopted as a periprocedural anticoagulant strategy for patients undergoing AF ablation. Meanwhile, there is growing evidence regarding the efficacy and safety of DOACs in patients with AF [53,54,55,56]. Recently, worldwide RCTs have revealed that UI-DOAC may be equivalent to UI-VKA [39,41,43,44]. Therefore, the latest guidelines classify the UI-DOAC strategy as a class I recommendation [8,9]. However, I-DOAC is also used as a periprocedural anticoagulant strategy in clinical practice owing to concerns regarding bleeding complications [20]. Although the HRS/EHRA/ECAS/APHRS/SOLAECE 2017 expert consensus statement on CA of AF classified the I-DOAC strategy as a class IIa recommendation [9], RCTs published after 2017 have demonstrated that there were no significant differences between the UI-DOAC and I-DOAC for the prevention of thromboembolic and major bleeding complications [46,47,48,49,50,51].

Thromboembolism is the most notable complication of CA for AF. The occurrence of periprocedural stroke or TIA was reported to be 0.1–0.6% in the latest guidelines [8]. Herein, both the DOAC and UI-VKA strategies revealed comparable efficacy in preventing thromboembolic events compared with I-VKA, and there were no significant differences among them. Therefore, an uninterrupted anticoagulant strategy is usually favorable, but an interrupted DOAC administration is feasible for the prevention of thromboembolism.

The safety of anticoagulants during periprocedural management must be carefully considered. Our NMA revealed a significant reduction in major bleeding complications with UI-DOAC compared with UI-VKA, consistent with the results of a previous meta-analysis [18]. The mechanism of reduction may be related to the type of anticoagulant (thrombin or factor Xa inhibitor) and a shorter half-life than warfarin. However, a recent meta-analysis showed no significant differences between UI-DOAC and UI-VKA [19]. In the sensitivity analysis, which excluded individual studies, we could not identify the robustness of UI-DOAC for significant reduction of major bleeding without each of the 10 studies in Table S5b. However, the estimated ORs with both DOAC strategies tended to carry a lower risk of major bleeding; thus, they may be safer alternatives to UI-VKA.

The ACE associated with CA for AF is relatively common and reported in 0–12.5% of UI-DOAC, 15.0–35.7% of I-DOAC, and 8.7–18.6% of UI-VKA cases [57,58,59]. In a previous meta-analysis, UI-DOAC significantly reduced the occurrence of ACE compared to I-DOAC [60]. In our review, similar ACE incidence rates were observed (UI-DOAC, 16.0%; I-DOAC, 20.7%; and UI-VKA, 15.4%), but we were unable to identify a significant reduction with UI-DOAC. Although ACE is classified as a complication of unknown significance in the current guidelines [8], it may be associated with the risk of dementia, cognitive impairment, and future stroke [61,62]. Nakamura et al. reported ACE detected post CA on follow-up MRI disappeared in 79.8% of cases [48]. The remaining 20.2% may develop chronic infarcts due to debris dislodging, air embolism, or small thrombosis [63,64]. The significance of ACE remains unclear, but a continuous anticoagulant strategy is feasible as a periprocedural treatment for ACE prevention.

We set another network with each anticoagulant regimen as a subgroup analysis and found that UI-dabigatran could significantly reduce the composite of the primary outcomes. Since UI-dabigatran did not influence significantly for thromboembolic events in Table S6b, the reduction of major bleeding complications can lead to this result. Although the DOACs included apixaban, dabigatran, edoxaban, and rivaroxaban, dabigatran is a thrombin inhibitor, while the others are factor Xa inhibitors. Dabigatran can extend the activated thromboplastin time, activated coagulation time (ACT), and thrombin time to a greater extent than factor Xa inhibitors [8]. Recent RCTs that compared UI-DOAC and UI-VKA revealed that the total amount of unfractionated heparin (UFH) during AF ablation increased, owing to the use of factor Xa inhibitors (apixaban, 156% [40]; edoxaban, 124% [44]; and rivaroxaban, 133% [39,42]) compared with thrombin inhibitors (dabigatran, 104% [41]), and the mean ACT was lower with factor Xa inhibitors than with thrombin inhibitors (307 vs. 330 s). This previously reported finding [65] may contribute to the increased risk of major bleeding. Martin et al. reported that ACT was strongly correlated with the prothrombin time-international normalized ratio and dabigatran concentration, but not with factor Xa inhibitor concentration [66]. Moreover, only dabigatran was parallel with VKA in the UFH dose–response curves. In contrast, factor Xa inhibitors had a smaller effect on ACT prolongation in response to heparin. The target ACT at 300 s is supported by robust evidence for controlling the thromboembolic and bleeding risks, but this evidence depends on VKA and UFH management [67]. Consequently, dabigatran is the optimal periprocedural anticoagulant for ACT monitoring during AF ablation.

As DOACs have become the practical standard for periprocedural anticoagulant strategies, the management of major bleeding is more important. Idarucizumab, a specific reversal agent for dabigatran, is now available worldwide [68]. In contrast, andexanet alfa, a specific reversal agent for factor Xa inhibitors, is only available in some countries [69]. Therefore, UI-dabigatran allows an option to manage complications if emergency bleeding occurs anywhere in the world.

Although NMA can assess the relative effectiveness of different strategies, our study has limitations. A primary limitation is that this NMA was based on study-level rather than patient-level data, which would considerably weaken the comparison validity. Second, differentiations of bleeding criteria, the usage of protamine and intracardiac echocardiography (ICE), lengths of follow-up, and methods of measuring ACE with MRI may contribute to heterogeneity and potentially affect the interpretation of the results. In particular, the number of participants who underwent MRI was limited in three RCTs. Further studies are needed to determine the significance of the optimal anticoagulant strategy for ACE. Additionally, the usage of protamine after the ablation procedure, and ICE during transseptal puncture, can prevent bleeding events. However, there were few studies to report those applications, and this can influence bleeding outcomes. Since four studies that investigated DOACs were not randomized into individual anticoagulants, a pooled comparison of a specific regimen in NMA could not be performed, and this weakened the interpretation of the results. Moreover, some regimens lacked data and were dependent on one study because of the limited number of RCTs. As RCTs of I-DOAC were mainly conducted in Japan, their results may involve regional bias.

## 5. Conclusions

In patients undergoing AF ablation, both DOAC strategies were associated with a lower incidence of major bleeding and had a similar effect on the prevention of thromboembolic events and minor bleeding compared with the UI-VKA strategy, whereas the I-VKA strategy should generally be avoided. Continuous DOAC and VKA administration was associated with a lower incidence of ACE. Therefore, UI-DOAC is the preferable alternative to UI-VKA. Although further data on the outcomes of patients receiving UI-dabigatran are needed for definitive conclusions, our results support the use of UI-dabigatran as the optimal periprocedural anticoagulant for ACT monitoring during AF ablation.

## Figures and Tables

**Table 1:** Baseline characteristics in the included studies.

Study	Year	Regimen	*n*	Age (years)	Male Sex	Paroxysmal AF	CHA_2_DS_2_-VASc	HAS-BLED	Mean ACT	Target ACT	Total UFH Dose	Protamine	ICE	Ablation Technology	Follow-Up Period
COMPARE [36] (International)	2014	UI-Warfarin	794	59	230 (73%)	200 (63%)	NR	NR	NR	>300	NR	Used ^†^	794 (100%)	RF	48 h
		I-Warfarin	790	59	245 (77%)	229 (72%)				>350			790 (100%)		
Nin [37] (Japan)	2013	I-Dabigatran 110 mg BID	45	61	38 (84%)	34 (76%)	NR	NR	NR	300–400	NR	Used ^†^	NR	RF	14 days
		I-Warfarin	45	61	36 (80%)	32 (71%)									
ABRIDGE-J [38] (Japan)	2019	I-Dabigatran 150/110 mg BID	220	65	171 (78%)	138 (63%)	2.0 *	1.0 *	NR	300–400	14,000	Used ^†^	52 (24%)	RF/Cryo	3 months
		UI-Warfarin	222	66	160 (72%)	138 (62%)	2.0 *	1.0 *			9000		58 (26%)		
VENTURE-AF [39] (International)	2015	UI-Rivaroxaban 20 mg OD	114	59	86 (75%)	95 (83%)	1.5	NR	302	300–400	13,871	32 (28%)	Used ^†^	Unclear	30 days
		UI-Warfarin	107	61	90 (84%)	87 (81%)	1.7		332		10,964	27 (25%)			
Kuwahara [40] (Japan)	2016	UI-Apixaban 5/2.5 mg BID	100	65	75 (75%)	59 (59%)	2.1	NR	322	>300	14,000	Used ^†^	NR	RF	7 days
		UI-Warfarin	100	66	72 (72%)	60 (60%)	2.4		357		9000				
RE-CIRCUIT [41] (International)	2017	UI-Dabigatran 150 mg BID	317	59	230 (73%)	213 (67%)	2.0	NR	330	>300	12,402	Used ^†^	NR	Mixed	56 days
		UI-Warfarin	318	59	245 (77%)	219 (69%)	2.2		342		11,910				
ASCERTAIN [42] (Japan)	2018	UI-Rivaroxaban 15/10 mg OD	64	59	53 (83%)	40 (63%)	NR	NR	299	300–350	12,500	Used ^†^	NR	RF	30 days
		UI-Warfarin	63	62	53 (84%)	42 (67%)			341		9000				
AXAFA-AFNET 5 [43] (International)	2018	UI-Apixaban 5/2.5 mg BID	318	64	218 (69%)	189 (59%)	2.4	NR	310	>300	NR	Used ^†^	Used ^†^	Mixed	3 months
		UI-Warfarin	315	64	206 (65%)	178 (57%)	2.4		349						
ELIMINATE-AF [44] (International)	2019	UI-Edoxaban 60 mg OD	375	60	290 (77%)	284 (76%)	1.8	NR	303	300–400	14,261	NR	92 (25%)	RF/Cryo	90 days
		UI-Warfarin	178	61	149 (84%)	131 (74%)	1.7		338		11,473		42 (24%)		
Yoshimura [45] (Japan)	2017	UI-Rivaroxaban 15/10 mg OD	55	59	45 (82%)	33 (60%)	1.7	NR	275	>300	15,745	NR	NR	RF	Unclear
		I-Apixaban 5/2.5 mg BID	50	59	41 (82%)	31 (62%)	1.7		286		14,240				
AEIOU [46] (USA)	2018	UI-Apixaban 5 mg BID	150	63	101 (67%)	100 (67%)	2.2	1.0	NR	>300	17,800	137 (91%)	NR	RF/Cryo	30 days
		I-Apixaban 5/2.5 mg BID	145	64	97 (67%)	91 (63%)	2.4	1.1			19,700	128 (88%)			
Yu [47] (Korea)	2019	UI-DOAC (Api/Dab/Riv)	106	59	81 (76%)	67 (63%)	1.6	NR	352	350–400	18,740	NR	Used ^†^	RF	1 month
		I-DOAC (Api/Dab/Riv)	110	58	79 (72%)	74 (67%)	1.7		348		20,136				
Nakamura [48] (Japan)	2019	UI-DOAC (Api/Dab/Edo/Riv)	421	65	298 (71%)	222 (53%)	2.0	1.3	358	300–400	12,936	405 (96%)	NR	RF	30 days
		I-DOAC (Api/Dab/Edo/Riv)	423	65	298 (70%)	236 (58%)	2.1	1.4	330		13,830	371 (88%)			
Nagao [49] (Japan)	2019	UI-DOAC (Api/Edo/Riv)	100	70	64 (64%)	57 (57%)	2.8	NR	285	>300	8704	Used ^†^	Used ^†^	RF	1 month
		I-DOAC (Api/Edo/Riv)	100	70	62 (62%)	59 (59%)	2.6		280		9945				
Ando [50] (Japan)	2019	UI-Apixaban 5 mg BID	32	67	26 (81%)	32 (100%)	NR	NR	NR	300–350	NR	Used ^†^	NR	Cryo	30 days
		I-Apixaban 5 mg BID	65	66	49 (75%)	65 (100%)									
Yamaji [51] (Japan)	2019	UI-DOAC (Api/Dab/Edo/Riv)	277	66	211 (76%)	171 (62%)	1.9	1.4	NR	300–400	NR	Used ^†^	NR	RF	90 days
		I-DOAC (Api/Dab/Edo/Riv)	307	65	212 (69%)	199 (65%)	1.9	1.4							
Yoshimoto [52] (Japan)	2021	UI-Edoxaban 60/30 mg OD	61	62	43 (70%)	38 (62%)	1.7	1.1	300	>300	7333	NR	NR	RF	Unclear
		UI-Rivaroxaban 15/10 mg OD	63	62	46 (73%)	45 (71%)	1.8	1.2	298		7865				

CHA_2_DS_2_-VASc and HAS-BLED scores are the risk prediction scores of stroke and major bleeding, respectively. AF, atrial fibrillation; ACT, activated coagulation time; UFH, unfractionated heparin; ICE, intracardiac echocardiography; UI, uninterrupted; I, interrupted; DOAC, direct anticoagulant; Api, apixaban; Dab, dabigatran; Edo, edoxaban; Riv, rivaroxaban; OD, omni die (once a day); BID, bis in die (twice a day); NR, not reported; RF, radiofrequency ablation; Cryo, cryoballoon ablation. * Median. ^†^ Numbers were unclear.

**Table 2:** P-score and the SUCRA values for each strategy and outcome.

Strategy	Thromboembolic Events		Major Bleeding		Composite of Primary Outcomes		Minor Bleeding		Asymptomatic Cerebral Embolism	
	P-Score	SUCRA	P-Score	SUCRA	P-Score	SUCRA	P-Score	SUCRA	P-Score	SUCRA
UI-DOAC	0.72	0.73	0.81	0.76	0.82	0.82	0.62	0.65	0.64	0.60
I-DOAC	0.68	0.70	0.82	0.85	0.77	0.77	0.52	0.49	0.07	0.09
UI-VKA	0.60	0.57	0.33	0.34	0.41	0.40	0.87	0.86	0.79	0.82
I-VKA	0.00	0.00	0.04	0.05	0.00	0.00	0.00	0.00	-	-

SUCRA, surface under the cumulative ranking; UI, uninterrupted; I, interrupted; DOAC, direct oral anticoagulant; VKA, vitamin-K antagonist.

**Table 3:** P-score and the SUCRA values for each strategy and composite of primary outcome.

Strategy	Composite of Primary Outcomes	
	P-Score	SUCRA
UI-dabigatran	0.93	0.95
I-dabigatran	0.89	0.82
UI-apixaban	0.52	0.53
I-apixaban	0.51	0.52
UI-VKA	0.46	0.47
UI-rivaroxaban	0.36	0.41
UI-edoxaban	0.33	0.30
I-VKA	0.00	0.00

SUCRA, surface under the cumulative ranking; UI, uninterrupted; I, interrupted; VKA, vitamin-K antagonist.

## Data Availability

All data are incorporated into the article and its online supplementary material.

## Supplementary Materials

The following supporting information can be downloaded at: https://www.mdpi.com/article/10.3390/jcm11071872/s1, Figure S1: Inconsistency analysis; Figure S2: Comparison adjusted funnel plot; Figure S3: Network of anticoagulant comparisons for the composite of primary outcomes; Table S1: PRISMA network meta-analysis checklist; Table S2: PICOS format and detailed search code; Table S3: Efficacy and safety outcomes in the included studies; Table S4: Assessment of bias in the randomized clinical trials; Table S5: Sensitivity analysis; Table S6: Summary estimates for outcomes with each anticoagulant regimen from network meta-analysis.
