# Supplementary material for: Optimal Anticoagulant Strategy for Periprocedural Management of Atrial Fibrillation Ablation: A Systematic Review and Network Meta-Analysis
Source: J Clin Med. 2022 Mar 28;11(7):1872. doi: 10.3390/jcm11071872 (PMC8999346; doi:10.3390/jcm11071872)
Supplement: Supplementary file 1 [file jcm-11-01872-s001.zip › jcm-1602656-supplementary.pdf]

Supplementary Data

Optimal anticoagulant strategy for periprocedural management of atrial fibrillation ablation: A systematic review and network meta-analysis

Tabito Kino, Minako Kagimoto, Takayuki Yamada, Satoshi Ishii, Masanari Asai, Shunichi Asano, Hideto Yano, Toshiyuki Ishikawa, and Tomoaki Ishigami

Supplementary Figures

Figure S1. Inconsistency analysis. 2

Figure S2. Comparison adjusted funnel plot. 3

Figure S3. Network of anticoagulant comparisons for the composite of primary outcomes. 4

Supplementary Tables

Table S1. PRISMA network meta-analysis checklist. 5

Table S2. PICOS format and detailed search code. 8

Table S3. Efficacy and safety outcomes in the included studies. 10

Table S4. Assessment of bias in the randomized clinical trials. 11

Table S5. Sensitivity analysis (leave-one-out study). 12

Table S6. Summary estimates for outcomes with each anticoagulant regimen from network meta-analysis. 14

Figure S1. Inconsistency analysis.

Inconsistency between direct and indirect evidence was examined (I) globally by running the design-by-treatment interaction model and (II) locally by using node-splitting analyses. Asymptomatic cerebral embolism could not be assessed because it did not have closed loop networks.

(I) The design-based decomposition of Cochran's Q showed that there was no inconsistency examined globally. (a) Thromboembolic events (Q = 1.52, df = 2, p = 0.47); (b) major bleeding (Q = 1.63, df = 1, p = 0.20); (c) composite of primary outcomes (Q = 3.41, df = 2, p = 0.18); and (d) minor bleeding (Q = 1.38, df = 2, p = 0.50).

(II) Node-splitting and inconsistency plot tests demonstrated that none of the comparisons were inconsistent. (a) Thromboembolic events; (b) major bleeding; (c) composite of primary outcomes; and (d) minor bleeding.

(a) Thromboembolic events
(Stroke, TIA, or systemic embolism)

Comparison	Number of Studies	Direct Evidence	OR	95%CI
**I-DOAC vs I-VKA**				
Direct estimate	1	0.35	0.33	[0.01; 8.22]
Indirect estimate			0.04	[0.00; 0.42]
Network estimate			0.08	[0.01; 0.56]
**I-DOAC vs UI-DOAC**				
Direct estimate	3	0.74	1.37	[0.26; 7.30]
Indirect estimate			1.34	[0.08; 23.17]
Network estimate			1.36	[0.32; 5.77]
**I–DOAC vs UI-VKA**				
Direct estimate	1	0.27	0.33	[0.01; 8.26]
Indirect estimate			2.15	[0.31; 14.83]
Network estimate			1.31	[0.25; 6.86]
**I-VKA vs UI-VKA**				
Direct estimate	1	0.87	20.58	[4.95; 85.56]
Indirect estimate			2.55	[0.06; 106.15]
Network estimate			15.77	[4.16; 59.70]
**UI-DOAC vs UI-VKA**				
Direct estimate	4	0.77	0.97	[0.20; 4.72]
Indirect estimate			0.95	[0.05; 17.36]
Network estimate			0.97	[0.24; 3.87]

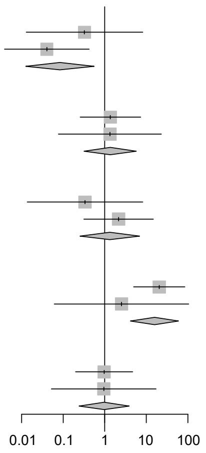

(c) Composite of primary outcomes
(Thromboembolic events and major bleeding)

Comparison	Number of Studies	Direct Evidence	OR	95%CI
**I-DOAC vs I-VKA**				
Direct estimate	1	0.19	0.33	[0.01; 9.38]
Indirect estimate			0.06	[0.01; 0.28]
Network estimate			0.08	[0.02; 0.33]
**I-DOAC vs UI-DOAC**				
Direct estimate	7	0.77	1.27	[0.52; 3.09]
Indirect estimate			0.53	[0.11; 2.68]
Network estimate			1.04	[0.48; 2.26]
**I-DOAC vs UI-VKA**				
Direct estimate	1	0.32	0.24	[0.05; 1.18]
Indirect estimate			0.99	[0.33; 2.94]
Network estimate			0.63	[0.26; 1.54]
**I-VKA vs UI-VKA**				
Direct estimate	1	0.88	9.98	[2.68; 37.15]
Indirect estimate			1.73	[0.05; 56.51]
Network estimate			8.02	[2.35; 27.45]
**UI-DOAC vs UI-VKA**				
Direct estimate	6	0.85	0.69	[0.34; 1.41]
Indirect estimate			0.29	[0.05; 1.58]
Network estimate			0.61	[0.31; 1.17]

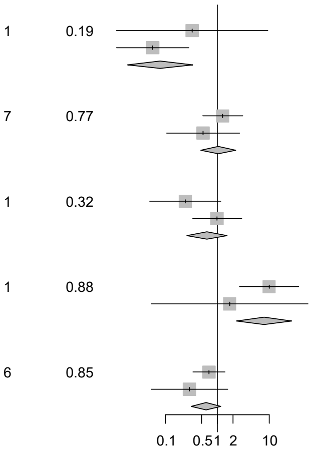

(b) Major bleeding

Comparison	Number of Studies	Direct Evidence	OR	95%CI
**I-DOAC vs UI-DOAC**				
Direct estimate	7	0.74	1.27	[0.52; 3.11]
Indirect estimate			0.42	[0.09; 1.92]
Network estimate			0.96	[0.44; 2.06]
**I-DOAC vs UI-VKA**				
Direct estimate	1	0.38	0.27	[0.07; 1.06]
Indirect estimate			0.79	[0.27; 2.33]
Network estimate			0.53	[0.22; 1.23]
**UI-DOAC vs UI-VKA**				
Direct estimate	6	0.88	0.62	[0.34; 1.14]
Indirect estimate			0.21	[0.04; 1.08]
Network estimate			0.55	[0.31; 0.97]

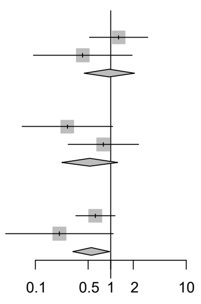

(d) Minor bleeding

Comparison	Number of Studies	Direct Evidence	OR	95%CI
**I-DOAC vs I-VKA**				
Direct estimate	1	0.28	0.31	[0.12; 0.80]
Indirect estimate			0.16	[0.09; 0.30]
Network estimate			0.20	[0.12; 0.33]
**I-DOAC vs UI-DOAC**				
Direct estimate	5	0.83	0.98	[0.66; 1.46]
Indirect estimate			1.68	[0.70; 4.03]
Network estimate			1.08	[0.75; 1.54]
**I-DOAC vs UI-VKA**				
Direct estimate	1	0.07	1.35	[0.30; 6.11]
Indirect estimate			1.18	[0.77; 1.80]
Network estimate			1.19	[0.79; 1.79]
**I-VKA vs UI-VKA**				
Direct estimate	1	0.88	6.51	[4.42; 9.60]
Indirect estimate			3.43	[1.22; 9.69]
Network estimate			6.02	[4.19; 8.66]
**UI-DOAC vs UI-VKA**				
Direct estimate	6	0.93	1.07	[0.84; 1.37]
Indirect estimate			1.82	[0.72; 4.63]
Network estimate			1.11	[0.87; 1.40]

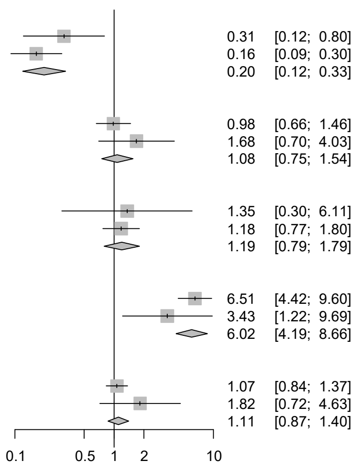

TIA, transient ischemic attack; UI, uninterrupted; I, interrupted; DOAC, direct oral anticoagulant; VKA, vitamin-K antagonist; OR, odds ratio; CI, confidence interval.

Figure S2. Comparison adjusted funnel plot.

Publication bias was analysed using Begg's rank correlation test and Egger's linear regression test. Overall, there was no significant publication bias. In asymptomatic cerebral embolism, both tests were omitted because of the number of studies. (a) Thromboembolic events (Begg's: $p = 0.65$ and Egger's: $p = 0.77$); (b) major bleeding (Begg's: $p = 0.52$ and Egger's: $p = 0.43$); (c) composite of primary outcomes (Begg's: $p = 0.72$ and Egger's: $p = 0.62$); and (d) minor bleeding (Begg's: $p = 0.48$ and Egger's: $p = 0.33$).

(a) Thromboembolic events
(Stroke, TIA, or systemic embolism)

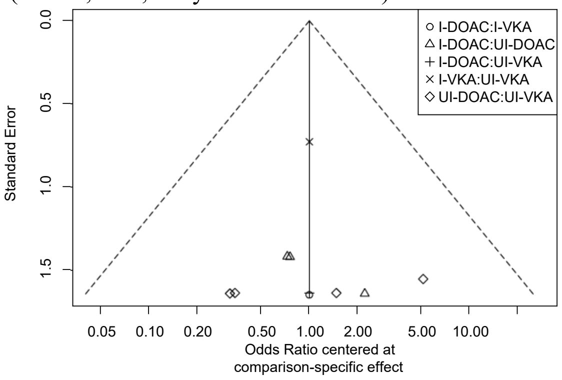

(c) Composite of primary outcomes
(Thromboembolic events and major bleeding)

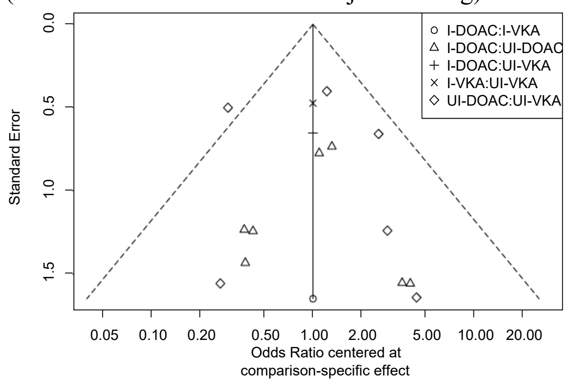

(b) Major bleeding

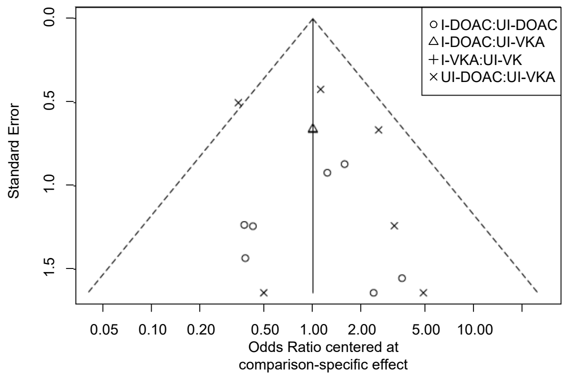

(d) Minor bleeding

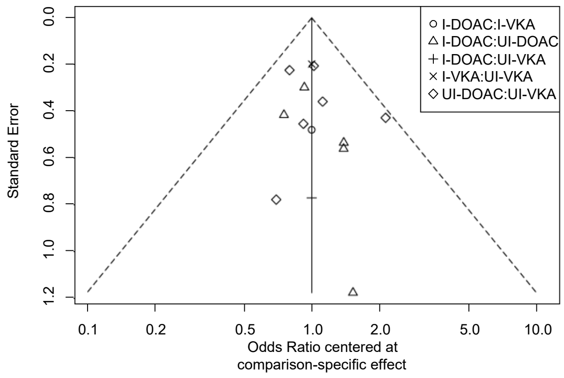

(e) Asymptomatic cerebral embolism

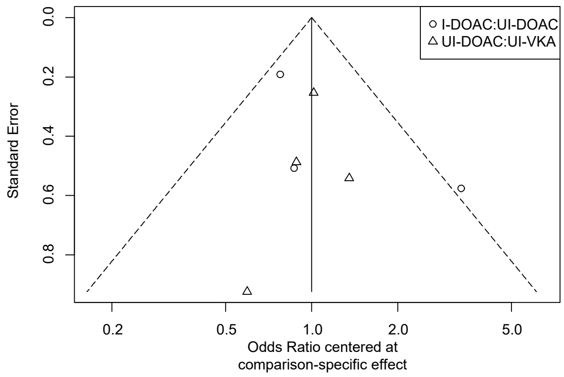

TIA, transient ischemic attack; UI, uninterrupted; I, interrupted; DOAC, direct oral anticoagulant; VKA, vitamin-K antagonist.

Figure S3. Network of anticoagulant comparisons for the composite of primary outcomes.

Directly comparable treatments are linked to lines. The nodes were placed and labelled according to the treatments. The thickness of the edges is proportional to the inverse standard error of the treatment effects, aggregated over all studies, including the two respective treatments. The network included 13 two-armed studies.

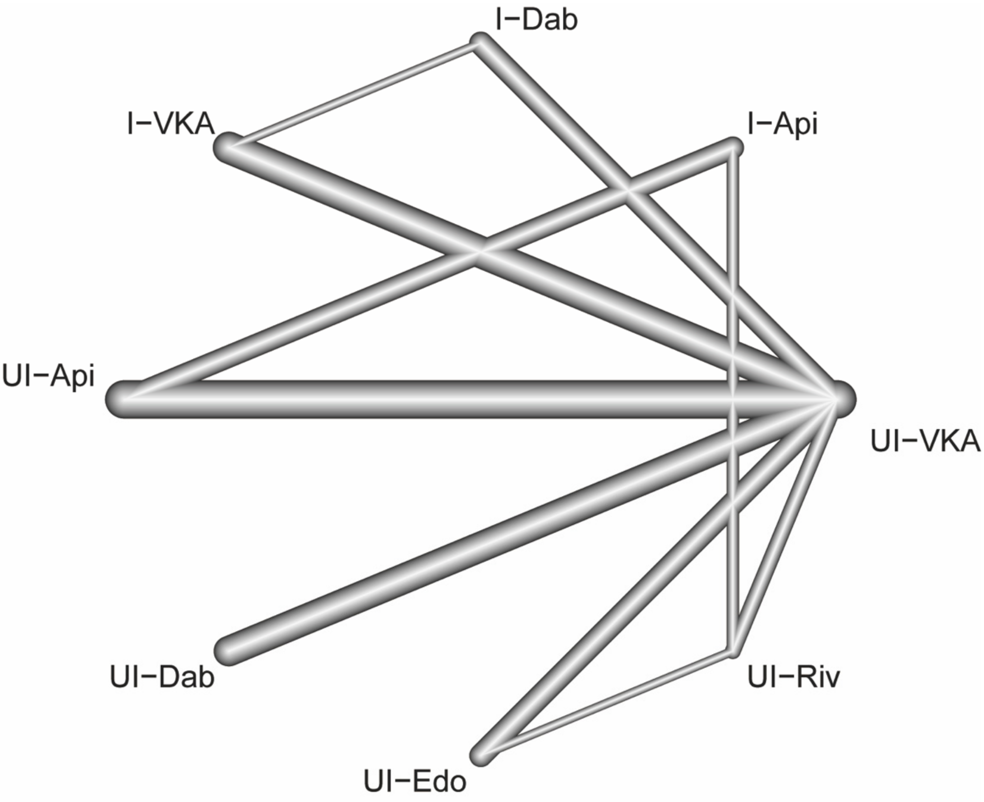

UI, uninterrupted; I, interrupted; Api, apixaban; Dab, dabigatran; Edo, edoxaban; Riv, rivaroxaban; VKA, vitamin-K antagonist.

Table S1. PRISMA network meta-analysis checklist of items to include when reporting a systematic review involving a network meta-analysis.

Section/Topic	Item #	Checklist Item	Reported on Page #
**TITLE**			
Title	1	Identify the report as a systematic review *incorporating a network meta-analysis (or related form of meta-analysis).*	1
**ABSTRACT**			
Structured summary	2	Provide a structured summary including, as applicable:<br>**Background:** main objectives<br>**Methods:** data sources; study eligibility criteria, participants, and interventions; study appraisal; and *synthesis methods, such as network meta-analysis.*<br>**Results:** number of studies and participants identified; summary estimates with corresponding confidence/credible intervals; *treatment rankings may also be discussed. Authors may choose to summarize pairwise comparisons against a chosen treatment included in their analyses for brevity.*<br>**Discussion/Conclusions:** limitations; conclusions and implications of findings.<br>**Other:** primary source of funding; systematic review registration number with registry name.	1
**INTRODUCTION**			
Rationale	3	Describe the rationale for the review in the context of what is already known, *including mention of why a network meta-analysis has been conducted.*	1-2
Objectives	4	Provide an explicit statement of questions being addressed, with reference to participants, interventions, comparisons, outcomes, and study design (PICOS).	2
**METHODS**			
Protocol and registration	5	Indicate whether a review protocol exists and if and where it can be accessed (e.g., Web address); and, if available, provide registration information, including registration number.	2
Eligibility criteria	6	Specify study characteristics (e.g., PICOS, length of follow-up) and report characteristics (e.g., years considered, language, publication status) used as criteria for eligibility, giving rationale. *Clearly describe eligible treatments included in the treatment network, and note whether any have been clustered or merged into the same node (with justification).*	2<br>Table S2
Information sources	7	Describe all information sources (e.g., databases with dates of coverage, contact with study authors to identify additional studies) in the search and date last searched.	2<br>Table S2
Search	8	Present full electronic search strategy for at least one database, including any limits used, such that it could be repeated.	2<br>Table S2
Study selection	9	State the process for selecting studies (i.e., screening, eligibility, included in systematic review, and, if applicable, included in the meta-analysis).	2
Data collection process	10	Describe method of data extraction from reports (e.g., piloted forms, independently, in duplicate) and any processes for obtaining and confirming data from investigators.	2-3

Section/Topic	Item #	Checklist Item	Reported on Page #
Data items	11	List and define all variables for which data were sought (e.g., PICOS, funding sources) and any assumptions and simplifications made.	2-3<br>Table S3
**Geometry of the network**	**S1**	Describe methods used to explore the geometry of the treatment network under study and potential biases related to it. This should include how the evidence base has been graphically summarized for presentation, and what characteristics were compiled and used to describe the evidence base to readers.	3<br>Figure 2
Risk of bias within individual studies	12	Describe methods used for assessing risk of bias of individual studies (including specification of whether this was done at the study or outcome level), and how this information is to be used in any data synthesis.	3
Summary measures	13	State the principal summary measures (e.g., risk ratio, difference in means). *Also describe the use of additional summary measures assessed, such as treatment rankings and surface under the cumulative ranking curve (SUCRA) values, as well as modified approaches used to present summary findings from meta-analyses.*	3
Planned methods of analysis	14	Describe the methods of handling data and combining results of studies for each network meta-analysis. This should include, but not be limited to:<br>• *Handling of multi-arm trials;*<br>• *Selection of variance structure;*<br>• *Selection of prior distributions in Bayesian analyses; and*<br>• *Assessment of model fit.*	3
**Assessment of Inconsistency**	**S2**	Describe the statistical methods used to evaluate the agreement of direct and indirect evidence in the treatment network(s) studied. Describe efforts taken to address its presence when found.	3
Risk of bias across studies	15	Specify any assessment of risk of bias that may affect the cumulative evidence (e.g., publication bias, selective reporting within studies).	3
Additional analyses	16	Describe methods of additional analyses if done, indicating which were pre-specified. This may include, but not be limited to, the following:<br>• Sensitivity or subgroup analyses;<br>• Meta-regression analyses;<br>• *Alternative formulations of the treatment network; and*<br>• *Use of alternative prior distributions for Bayesian analyses (if applicable).*	3
**RESULTS**			
Study selection	17	Give numbers of studies screened, assessed for eligibility, and included in the review, with reasons for exclusions at each stage, ideally with a flow diagram.	3-4<br>Figure 1
**Presentation of network structure**	**S3**	Provide a network graph of the included studies to enable visualization of the geometry of the treatment network.	7<br>Figure 2
**Summary of network geometry**	**S4**	Provide a brief overview of characteristics of the treatment network. This may include commentary on the abundance of trials and randomized patients for the different interventions and pairwise comparisons in the network, gaps of evidence in the treatment network, and potential biases reflected by the network structure.	7<br>Figure 2

Section/Topic	Item #	Checklist Item	Reported on Page #
Study characteristics	18	For each study, present characteristics for which data were extracted (e.g., study size, PICOS, follow-up period) and provide the citations.	4<br>Table 1
Risk of bias within studies	19	Present data on risk of bias of each study and, if available, any outcome level assessment.	7<br>Table S4
Results of individual studies	20	For all outcomes considered (benefits or harms), present, for each study: 1) simple summary data for each intervention group, and 2) effect estimates and confidence intervals. Modified approaches may be needed to deal with information from larger networks.	4<br>Table S3
Synthesis of results	21	Present results of each meta-analysis done, including confidence/credible intervals. *In larger networks, authors may focus on comparisons versus a particular comparator (e.g. placebo or standard care), with full findings presented in an appendix. League tables and forest plots may be considered to summarize pairwise comparisons*. If additional summary measures were explored (such as treatment rankings), these should also be presented.	7-9<br>Figure 3<br>Table 2
**Exploration for inconsistency**	**S5**	Describe results from investigations of inconsistency. This may include such information as measures of model fit to compare consistency and inconsistency models, P values from statistical tests, or summary of inconsistency estimates from different parts of the treatment network.	9<br>Figure S1
Risk of bias across studies	22	Present results of any assessment of risk of bias across studies for the evidence base being studied.	9<br>Figure S2
Results of additional analyses	23	Give results of additional analyses, if done (e.g., sensitivity or subgroup analyses, meta-regression analyses, *alternative network geometries studied, alternative choice of prior distributions for Bayesian analyses*, and so forth).	9-10<br>Figure 4, S3<br>Table 3, S5, S6
**DISCUSSION**			
Summary of evidence	24	Summarize the main findings, including the strength of evidence for each main outcome; consider their relevance to key groups (e.g., healthcare providers, users, and policy-makers).	10
Limitations	25	Discuss limitations at study and outcome level (e.g., risk of bias), and at review level (e.g., incomplete retrieval of identified research, reporting bias). *Comment on the validity of the assumptions, such as transitivity and consistency. Comment on any concerns regarding network geometry (e.g., avoidance of certain comparisons).*	11-12
Conclusions	26	Provide a general interpretation of the results in the context of other evidence, and implications for future research.	12
**FUNDING**			
Funding	27	Describe sources of funding for the systematic review and other support (e.g., supply of data); role of funders for the systematic review. This should also include information regarding whether funding has been received from manufacturers of treatments in the network and/or whether some of the authors are content experts with professional conflicts of interest that could affect use of treatments in the network.	12

Text in italics indicates wording specific to the reporting of network meta-analyses that has been added to guidance from the PRISMA statement. PICOS: population, intervention, comparators, outcomes, study design.

Table S2. Detailed search strategy.

**(a).** PICOS format		
**PICOS format**		
P	Population	Patients with atrial fibrillation who received ablation treatment
I	Intervention	Periprocedural anticoagulant management
C	Comparators	Uninterrupted vitamin K antagonist
O	Outcomes	Thromboembolic events (stroke, transient ischemic attack, or systemic embolism), major bleeding, minor bleeding, and asymptomatic cerebral embolism
S	Study design	Randomized controlled trial

**(b).** Detailed search code in each database		
**MEDLINE**		
#	Query	Hits
1	("atrial fibrillation"[All Fields] OR ("atrial"[All Fields] AND "fibrillation"[All Fields]) OR "atrial fibrillation"[All Fields])	90735
2	("ablate"[All Fields] OR "ablated"[All Fields] OR "ablates"[All Fields] OR "ablating"[All Fields] OR "ablation"[All Fields] OR "ablational"[All Fields] OR "ablations"[All Fields])	120649
3	("periprocedural"[All Fields] OR "periprocedurally"[All Fields] OR "periprocedure"[All Fields]) AND ("anticoagulants"[Pharmacological Action] OR "anticoagulants"[MeSH Terms] OR "anticoagulants"[All Fields] OR "anticoagulant"[All Fields] OR "anticoagulate"[All Fields] OR "anticoagulated"[All Fields] OR "anticoagulating"[All Fields] OR "anticoagulation"[All Fields] OR "anticoagulations"[All Fields] OR "anticoagulative"[All Fields])	1210
4	#1 AND #2	17295
5	#3 AND #4	273
6	#5 AND ("randomized controlled trial"[Publication Type] OR "randomized controlled trials as topic"[All Fields] OR "randomized controlled trial"[All Fields] OR "randomised controlled trial"[All Fields])	20
**EMBASE**		
#	Query	Hits
1	'atrial fibrillation'/exp OR 'atrial fibrillation'	89579
2	'ablation'	96803
3	'periprocedural anticoagulation' OR (periprocedural AND ('anticoagulation'/exp OR anticoagulation))	637
4	'atrial fibrillation ablation'/exp OR 'atrial fibrillation ablation' OR (atrial AND ('fibrillation'/exp OR fibrillation) AND ablation)	13969
5	'atrial fibrillation ablation periprocedural anticoagulation' OR (atrial AND ('fibrillation'/exp OR fibrillation) AND ablation AND periprocedural AND ('anticoagulation'/exp OR anticoagulation))	188
6	'atrial fibrillation ablation procedural anticoagulation randomized controlled trial' OR (atrial AND ('fibrillation'/exp OR fibrillation) AND ablation AND procedural AND ('anticoagulation'/exp OR anticoagulation) AND randomized AND controlled AND ('trial'/exp OR trial))	34
**CENTRAL**		
#	Query	Hits
1	atrial fibrillation	13328
2	ablation	9943
3	periprocedural anticoagulation	228
4	#1 AND #2	2760
5	#3 AND #4	70
6	#5 AND randomized controlled trial	48

(Word variations have been searched)

Web of Science		
#	Query	Hits
1	ALL=(atrial fibrillation)	63795
2	ALL=(ablation)	138775
3	ALL=(periprocedural anticoagulation)	559
4	(ALL=(atrial fibrillation)) AND ALL=(ablation)	13628
5	((ALL=(atrial fibrillation)) AND ALL=(ablation)) AND ALL=(periprocedural anticoagulation)	198
6	(((ALL=(atrial fibrillation)) AND ALL=(ablation)) AND ALL=(periprocedural anticoagulation)) AND ALL=(randomized controlled trial)	22

(Word variations have been searched)

Table S3. Efficacy and safety outcomes in the included studies.

Study	Year	Regimen	n	Thrombo-embolic events	Major Bleeding Criteria	Major Bleeding n	Minor Bleeding Criteria	Minor Bleeding n	ACE Time to MRI	ACE n
COMPARE (International)	2014	UI-Warfarin	794	2	BARC	3	BARC	33	NR	NR
		I-Warfarin	790	39		8		174		
Nin (Japan)	2013	Dabigatran 110 mg BID	45	0	Requiring intervention	0	Not requiring intervention	9	NR	NR
		I-Warfarin	45	1		0		20		
ABRIDGE-J (Japan)	2019	I-Dabigatran 150/110 mg BID	220	0	ISTH	3	Not fulfil ISTH	4	NR	NR
		UI-Warfarin	222	1		11		3		
VENTURE-AF (International)	2015	UI-Rivaroxaban 20 mg OD	114	0	ISTH, GUSTO, or TIMI major bleeding	0	No fulfil Major bleeding	21	NR	NR
		UI-Warfarin	107	1		1		17		
Kuwahara (Japan)	2016	UI-Apixaban 5/2.5 mg BID	100	0	ISTH	1	Not fulfil ISTH	3	< 48 h	2
		UI-Warfarin	100	0		0		4		3
RE-CIRCUIT (International)	2017	UI-Dabigatran 150 mg BID	317	0	ISTH	5	Not fulfil ISTH	60	NR	NR
		UI-Warfarin	318	1		22		56		
ASCERTAIN (Japan)	2018	UI-Rivaroxaban 15/10 mg OD	64	0	Requiring intervention	2	Not requiring intervention	12	< 24 h	10
		UI-Warfarin	63	0		1		12		10
AXAFA-AFNET 5 (International)	2018	UI-Apixaban 5/2.5 mg BID	318	2	ISTH, BARC, or TIMI major bleeding	10	Not fulfil ISTH, BARC, or TIMI major bleeding	44	< 48 h	44
		UI-Warfarin	315	0		14		50		40
ELIMINATE-AF (International)	2019	UI-Edoxaban 60 mg OD	375	1	ISTH	10	Not fulfil ISTH	32	< 96 h	16
		UI-Warfarin	178	0		3		7		5
Yoshimura (Japan)	2017	UI-Rivaroxaban 15/10 mg OD	55	0	Hemopericardium requiring pericardiocentesis	2	NR	NR	< 24 h	9
		I-Apixaban 5/2.5 mg BID	50	0		1				10
AEIOU (USA)	2018	UI-Apixaban 5 mg BID	150	1	BARC	2	BARC	15	NR	NR
		I-Apixaban 5/2.5 mg BID	145	1		3		11		
Yu (Korea)	2019	UI-DOAC (Api/Dab/Riv)	106	0	ISTH	2	NR	NR	NR	NR
		I-DOAC (Api/Dab/Riv)	110	0		1				
Nakamura (Japan)	2019	UI-DOAC (Api/Dab/Edo/Riv)	421	1	Requiring intervention	2	Not requiring intervention	25	< 24 h	69
		I-DOAC (Api/Dab/Edo/Riv)	423	1		4		23		69
Nagao (Japan)	2019	UI-DOAC (Api/Edo/Riv)	100	0	Requiring intervention	0	Not requiring intervention	6	< 24 h	4
		I-DOAC (Api/Edo/Riv)	100	1		1		8		17
Ando (Japan)	2019	UI-Apixaban 5 mg BID	32	0	ISTH	1	Not fulfil ISTH	1	NR	NR
		I-Apixaban 5 mg BID	65	0		1		3		
Yamaji (Japan)	2019	UI-DOAC (Api/Dab/Edo/Riv)	277	0	Requiring intervention	0	Not requiring intervention	6	NR	NR
		I-DOAC (Api/Dab/Edo/Riv)	307	0		2		9		
Yoshimoto (Japan)	2021	UI-Edoxaban 60/30 mg OD	61	0	Hemopericardium requiring pericardiocentesis	0	NR	NR	< 24 h	12
		UI-Rivaroxaban 15/10 mg OD	63	0		1				5

ACE, asymptomatic cerebral embolism; MRI, magnetic resonance imaging; UI, uninterrupted; I, interrupted; DOAC, direct anticoagulant; Api, apixaban; Dab, dabigatran; Edo, edoxaban; Riv, rivaroxaban; OD, omni die (once a day); BID, bis in die (twice a day); ISTH, International Society on Thrombosis and Hemostasis; BARC, Bleeding Academic Research Consortium; GUSTO, Global Usage of Strategies to Open Occluded Arteries; TIMI, Thrombolysis in Myocardial Infarction; NR, not reported.

Table S4. Assessment of bias in the randomized clinical trials using the Cochrane Collaboration's Tool.

Study	Year	D1	D2	D3	D4	D5	Overall
COMPARE	2014	+	+	+	+	+	**Low risk**
Nin	2013	!	+	+	+	+	**Some concerns**
ABRIDGE-J	2019	+	!	+	+	+	**Some concerns**
VENTURE-AF	2015	+	!	!	+	+	**Some concerns**
Kuwahara	2016	+	+	+	+	+	**Low risk**
RE-CIRCUIT	2017	+	!	+	+	+	**Some concerns**
ASCERTAIN	2018	+	!	+	+	+	**Some concerns**
AXAFA-AFNET 5	2018	+	!	+	+	+	**Some concerns**
ELIMINATE-AF	2019	+	!	+	+	+	**Some concerns**
Yoshimura	2017	!	!	+	+	+	**Some concerns**
AEIOU	2018	+	!	+	+	+	**Some concerns**
Yu	2019	+	+	+	+	+	**Low risk**
Nakamura	2019	!	!	+	+	+	**Some concerns**
Nagao	2019	!	+	+	+	+	**Some concerns**
Ando	2019	!	+	+	+	+	**Some concerns**
Yamaji	2019	!	+	+	+	+	**Some concerns**
Yoshimoto	2021	!	!	+	+	+	**Some concerns**

D1, randomization process; D2, deviations from intended interventions; D3, missing outcome data; D4, measurement of the outcome; D5, selection of the reported result; +, low risk; !, some concerns; -, high risk.

Table S5. Sensitivity analysis (leave-one-out study).

**(a).** Composite of primary outcomes (thromboembolic events and major bleeding).									
**Excluded Study**		**UI-DOAC**		**I-DOAC**		**I-VKA**		***p***	***I^2***
		**Estimated OR**	**95% CI**	**Estimated OR**	**95% CI**	**Estimated OR**	**95% CI**		
COMPARE	2014	0.58	0.30-1.14	0.57	0.22-1.44	1.74	0.05-56.91	0.26	24.7%
Nin	2013	0.58	0.30-1.14	0.57	0.22-1.44	9.98	2.65-37.57	0.26	24.7%
ABRIDGE-J	2019	0.71	0.37-1.35	0.97	0.35-2.71	8.80	2.84-26.64	0.26	15.1%
VENTURE-AF	2015	0.64	0.32-1.29	0.66	0.26-1.66	7.99	2.25-28.40	0.23	26.8%
Kuwahara	2016	0.57	0.29-1.17	0.60	0.24-1.49	7.96	2.31-27.39	0.24	24.7%
RE-CIRCUIT	2017	0.84	0.48-1.48	0.76	0.35-1.63	8.93	3.66-21.81	0.81	0.0%
ASCERTAIN	2018	0.56	0.28-1.11	0.59	0.24-1.48	7.94	2.29-27.46	0.24	24.8%
AXAFA-AFNET 5	2018	0.54	0.24-1.22	0.58	0.22-1.56	7.78	2.12-28.54	0.23	23.3%
ELIMINATE-AF	2019	0.48	0.25-0.91	0.52	0.22-1.22	8.21	2.79-24.18	0.37	12.4%
Yoshimura	2017	0.60	0.30-1.20	0.67	0.26-1.74	7.95	2.18-28.98	0.22	28.1%
AEIOU	2018	0.62	0.31-1.26	0.61	0.23-1.63	7.79	2.09-29.03	0.19	28.4%
Yu	2019	0.60	0.30-1.20	0.68	0.26-1.75	7.98	2.21-28.84	0.23	27.6%
Nakamura	2019	0.63	0.31-1.25	0.58	0.22-1.54	7.79	2.14-28.32	0.20	26.7%
Nagao	2019	0.62	0.32-1.20	0.58	0.23-1.44	7.94	2.33-27.11	0.24	24.2%
Ando	2019	0.60	0.30-1.20	0.67	0.26-1.71	7.96	2.19-28.88	0.22	28.1%
Yamaji	2019	0.62	0.32-1.20	0.58	0.23-1.46	7.93	2.30-27.34	0.23	24.9%
Combined		0.61	0.31-1.17	0.63	0.26-1.54	8.02	2.35-27.45	0.26	23.4%
UI, uninterrupted; I, interrupted; DOAC, direct oral anticoagulant; VKA, vitamin-K antagonist; OR, odds ratio; CI, confidence interval.									

**(b).** Major bleeding.									
**Excluded Study**		**UI-DOAC**		**I-DOAC**		**I-VKA**		***p***	***I^2***
		**Estimated OR**	**95% CI**	**Estimated OR**	**95% CI**	**Estimated OR**	**95% CI**		
COMPARE	2014	0.55	0.31-0.97	0.53	0.22-1.23	-	-	0.41	7.8%
ABRIDGE-J	2019	0.61	0.35-1.08	0.78	0.28-2.23	2.70	0.69-10.62	0.41	3.3%
VENTURE-AF	2015	0.57	0.31-1.06	0.54	0.22-1.33	2.70	0.60-12.10	0.34	14.7%
Kuwahara	2016	0.52	0.29-0.92	0.51	0.22-1.18	2.70	0.66-11.07	0.41	7.4%

RE-CIRCUIT	2017	0.76	0.42-1.37	0.65	0.28-1.48	2.70	0.71-10.21	0.87	0.0%
ASCERTAIN	2018	0.51	0.29-0.91	0.50	0.21-1.17	2.70	0.66-11.06	0.41	7.1%
AXAFA-AFNET 5	2018	0.50	0.24-1.05	0.50	0.19-1.28	2.70	0.60-12.16	0.34	11.5%
ELIMINATE-AF	2019	0.45	0.26-0.77	0.45	0.20-1.01	2.70	0.71-10.21	0.55	0.0%
Yoshimura	2017	0.55	0.30-1.01	0.56	0.23-1.41	2.70	0.61-12.02	0.37	14.0%
AEIOU	2018	0.56	0.31-1.04	0.49	0.19-1.25	2.70	0.61-11.95	0.33	13.1%
Yu	2019	0.55	0.30-1.00	0.57	0.23-1.42	2.70	0.61-11.90	0.39	13.2%
Nakamura	2019	0.57	0.32-1.01	0.46	0.18-1.14	2.70	0.64-11.36	0.36	9.1%
Nagao	2019	0.56	0.31-1.01	0.50	0.21-1.22	2.70	0.62-11.68	0.35	11.9%
Ando	2019	0.55	0.30-1.01	0.56	0.23-1.39	2.70	0.61-11.99	0.37	13.9%
Yamaji	2019	0.56	0.32-0.98	0.48	0.20-1.14	2.70	0.65-11.12	0.39	7.8%
Combined		0.55	0.31-0.97	0.52	0.22-1.23	2.70	0.65-11.18	0.41	7.8%

UI, uninterrupted; I, interrupted; DOAC, direct oral anticoagulant; VKA, vitamin-K antagonist; OR, odds ratio; CI, confidence interval

Table S6. Summary estimates for outcomes with each anticoagulant regimen from network meta-analysis.

Odds ratio [95% confidence interval] between column and row treatment regimens are summarized. Odds ratio smaller than 1 means that the odds of having an event for the row treatment regimen is lower than the column treatment regimen. Statistically significant results, where the 95% confidence interval does not include 1, are in bold.

**(a).** Composite of primary outcomes (thromboembolic events and major bleeding).							
**UI-Dab**	0.61<br>[0.13-2.87]	**0.23**<br>**[0.07-0.79]**	0.23<br>[0.04-1.21]	**0.14**<br>**[0.03-0.67]**	**0.15**<br>**[0.03-0.87]**	**0.21**<br>**[0.08-0.55]**	**0.02**<br>**[0.01-0.09]**
1.64<br>[0.35-7.68]	**I-Dab**	0.38<br>[0.09-1.55]	0.37<br>[0.06-2.26]	0.23<br>[0.04-1.26]	0.25<br>[0.04-1.61]	0.34<br>[0.10-1.11]	**0.04**<br>**[0.01-0.17]**

**4.32 [1.26-14.78]**	2.64 [0.65-10.79]	**UI-Api**	0.97 [0.29-3.30]	0.61 [0.15-2.48]	0.65 [0.14-3.02]	0.89 [0.42-1.87]	**0.11 [0.03-0.34]**
4.43 [0.83-23.74]	2.71 [0.44-16.59]	1.03 [0.30-3.48]	**I-Api**	0.62 [0.11-3.69]	0.67 [0.13-3.53]	0.91 [0.23-3.56]	**0.11 [0.02- 0.55]**
**7.12 [1.50-33.89]**	4.35 [0.79-23.91]	1.65 [0.40-6.75]	1.61 [0.27-9.52]	**UI-Edo**	1.08 [0.19-6.06]	1.46 [0.44-4.93]	**0.17 [0.04- 0.79]**
**6.59 [1.15-37.67]**	4.03 [0.62-26.20]	1.53 [0.33-7.04]	1.49 [0.28-7.81]	0.93 [0.17-5.19]	**UI-Riv**	1.35 [0.32-5.72]	**0.16 [0.03- 0.88]**
**4.87 [1.83-12.97]**	2.97 [0.90-9.83]	1.13 [0.54-2.37]	1.10 [0.28-4.29]	0.68 [0.20-2.30]	0.74 [0.17-3.12]	**UI-VKA**	**0.12 [0.05- 0.29]**
**40.85 [10.83-154.12]**	**24.96 [6.04-103.17]**	**9.46 [2.95-30.32]**	**9.22 [1.81-47.07]**	**5.74 [1.27-25.91]**	**6.20 [1.14-33.80]**	**8.39 [3.43-20.56]**	**I-VKA**

UI, uninterrupted; I, interrupted; Dab, dabigatran; Api, apixaban; Edo, edoxaban; Riv, rivaroxaban; VKA, vitamin-K antagonist

**(b).** Thromboembolic events (Stroke, TIA, or systemic embolism).							
**UI-Dab**	0.29 [0.00-33.48]	0.07 [0.00-12.51]	0.06 [0.00-33.41]	0.23 [0.00-48.10]	1.08 [0.01-222.67]	0.33 [0.01-14.43]	0.03 [0.00- 2.16]
3.45 [0.03-398.65]	**I-Dab**	0.23 [0.00-23.90]	0.22 [0.00-70.90]	0.80 [0.01-92.99]	3.71 [0.03-430.68]	1.15 [0.06-20.72]	0.09 [0.01- 1.70]
14.95 [0.08-2798.60]	4.33 [0.04-448.94]	**UI-Api**	0.97 [0.03-29.41]	3.49 [0.02-652.77]	16.08 [0.09-3022.10]	4.98 [0.13-187.89]	0.41 [0.01-28.64]
15.47 [0.03-8000.25]	4.48 [0.01-1425.84]	1.03 [0.03-31.49]	**I-Api**	3.61 [0.01-1865.84]	16.64 [0.03-8633.05]	5.16 [0.04-753.23]	0.42 [0.00-98.52]
4.29 [0.02-885.28]	1.24 [0.01-143.75]	0.29 [0.00-53.73]	0.28 [0.00-143.42]	**UI-Edo**	4.61 [0.02-955.91]	1.43 [0.03-61.97]	0.12 [0.00- 9.26]
0.93 [0.00-192.63]	0.27 [0.00-31.29]	0.06 [0.00-11.69]	0.06 [0.00-31.19]	0.22 [0.00-44.93]	**UI-Riv**	0.31 [0.01-13.50]	0.03 [0.00- 2.02]
3.00 [0.07-129.91]	0.87 [0.05-15.67]	0.20 [0.01- 7.56]	0.19 [0.00-28.32]	0.70 [0.02-30.31]	3.23 [0.07-140.52]	**UI-VKA**	**0.08 [0.01- 0.75]**
36.79 [0.46-2918.95]	10.66 [0.59-192.86]	2.49 [0.03-173.33]	2.38 [0.01-556.84]	8.57 [0.11-680.93]	39.55 [0.50-3154.77]	**12.26 [1.33-112.97]**	**I-VKA**

TIA, transient ischemic attack; UI, uninterrupted; I, interrupted; Dab, dabigatran; Api, apixaban; Edo, edoxaban; Riv, rivaroxaban; VKA, vitamin-K antagonist
